# A national assessment of elective cesarean sections in Bangladesh and the need for health literacy and accessibility

**DOI:** 10.1038/s41598-021-96337-0

**Published:** 2021-08-19

**Authors:** Awan Afiaz, Anowara Rayhan Arusha, Nurjahan Ananna, Enamul Kabir, Raaj Kishore Biswas

**Affiliations:** 1 Institute of Statistical Research and Training, University of Dhaka, Dhaka, Bangladesh; 2 Applied Linguistics and English Language Teaching (ELT), University of Dhaka, Dhaka, Bangladesh; 3 Ibrahim Medical College, Dhaka, Bangladesh; 4 School of Sciences, University of Southern Queensland, Toowoomba, QLD Australia; 5 Transport and Road Safety (TARS) Research Centre, School of Aviation, University of New South Wales, Sydney, Australia

**Keywords:** Health services, Public health, Medical research

## Abstract

There has been a gradual rise in the number of cesarean sections (CSs) in Bangladesh. The present study identified the cohort of women, who were more likely to opt for an elective CS based on their sociodemographic characteristics, pre-delivery care history, and media exposure, using the Bangladesh Multiple Indicator Cluster Survey-2019. The survey stratification adjusted logistic regression model and interpretable machine learning method of building classification trees were utilized to analyze a sample of 9202 women, alongside district-wise heat maps. One-in-five births (20%) were elective CSs in the 2 years prior to the survey. Women residing in affluent households with educated house-heads, who accessed antenatal care prior to delivery (AOR 4.12; 95% CI 3.06, 5.54) with regular access to media (AOR 1.31; 95% CI 1.10, 1.56) and who owned a mobile phone (AOR 1.25; 95% CI 1.04, 1.50) were more likely to opt for elective CSs, which suggests that health access and health literacy were crucial factors in women’s mode of delivery. Spatial analyses revealed that women living in larger cities had more elective CS deliveries, pointing towards the availability of better health and access to multiple safe delivery options in peripheral areas.

## Introduction

Socioeconomic changes and scientific advancement have led to multidimensional changes in the birth delivery system and consequently a gradual increase in the prevalence of cesarean sections (CSs). For nearly 30 years, the international healthcare community has considered the ideal rate for CSs to be between 10 and 15%^1,2^, with a recently revised statement in 2014 from the World Health Organization stating that over 10% of CS in a population is not associated with a reduction in maternal and neonatal mortality rate^3,4^. However, the proportion of CS delivery has increased from 6.7% in 1990 to 21.1% in 2015 globally, with large variations across countries ranging from 0.4 to 40%^5–8^. While an increased number of studies focused on developed nations, there is a need for assessing this gradual increase in developing nations as well.

There are multifaceted impacts of increased CSs ranging from maternal and child health to the influence on the overall health system to the cost of healthcare. Studies reported that CS is associated with child and adolescent obesity, which translates to the risk of early life^9^. An association was detected for inflammatory bowel diseases^10^ and other autoimmune diseases such as asthma, allergies, type 1 diabetes, and celiac disease^11^. While these additional issues would create pressure on the health system^12^, in many cases CS deliveries are medically justified and can reduce the risk of maternal and neonatal mortality^13^. However, the increasing number of elective CS (defined later) requires the health system to be equipped for additional work. This entails undertaking training programs for health workers^14^, providing delivery facilities in remote areas, and allocating budget for CS^11^, all of which are financially and logistically challenging for many developing nations^15^. Such a scenario leads to the necessity of estimating the prevalence of deliveries by CS on a national scale and also require studying the sociodemographic determinants of those who consider opting for elective CS as opposed to a normal vaginal delivery.

Substantial increases in CS rates have been recorded in several South Asian countries over the past decades. In Bangladesh, rates rose from 2% (2000) to 17% (2011) and to 36% in 2019; in India, from 3% (1992) to 11% (2006); and in Nepal, from 1% (2000) to 5% (2011)^16–20^. Literature suggests that the rise in CSs is caused by the pressures of supply and demand: providers often have financial incentives for intervening surgically, and these combine with the tendency toward risk avoidance by women of higher socioeconomic status as well as increased media influence^6,21–23^. A recent analysis of combined data of the Demographic and Health Surveys of the 43 Asian and African countries revealed that the urban rich had a higher rate of CS while deceased prevalence was observed among rural poorer households^24^.

For this study, elective CS deliveries refer to deliveries that were driven by non-medical reasons and the decision to perform these deliveries was taken before the mother went into labor^13,25^. CS delivery can be considered a useful strategy for reducing both maternal and neonatal morbidity^13^. However, although there is evidence that CS deliveries could reduce the risk of some birth and reproductive health complications^13,26,27^, these deliveries were also found to be associated with increased postpartum depression among mothers^28^. However, given the literature gap regarding the determinants of elective CS in the lower-and-middle-income countries (LMICs), this literature review would refer to general CS where the type (elective or non-elective) is not specified.

Sociodemographic and economic factors contribute to the popularity and wider use of CS deliverers among women. Women in higher socioeconomic tiers were found to prefer CSs to normal vaginal births^29^. Despite economic constraints, women with greater decision-making power at home were more likely to allocate resources for medicalized care (antenatal and birth), particularly if they feared a lack of proper medical treatment during delivery^29^. CS was also found to be more common in well-educated and wealthy women with health insurance^30^. Multiple studies on Bangladesh also revealed a similar scenario of educated, affluent and urban women preferring CS deliveries to normal vaginal births^31,32^.

However, these studies do not differentiate between elective CS deliveries and medically warranted CS deliveries. With the development of healthcare facilities and access, it could be argued that the increase in CS deliveries as a whole is something to be expected. However, the increase in such deliveries not due to medically induced reasons dilute need to be studied separately from the overall CS related studies. Furthermore, many of these study data are outdated and most of these did not assess the geographical heterogeneity of elective CS deliveries in Bangladesh, as was done in this study. Furthermore, the current study explored the effect of the history of pre-delivery care and media exposure on elective CS deliveries in addition to the typical sociodemographic variables considered in other studies.

The use of mobiles phones and access to the internet are considered to be major contributors to the increased CS rate in recent years. Around 99% of US women, who had given birth in 2012, used electronic devices (laptops, desktops, smartphones, tablets) at least once in the previous week, and they reported these devices as an excellent way to find information about pregnancy and childbirth^33^. A follow-up study revealed that two-in-three pregnant women in the U.S. who chose CS deliveries received regular email updates regarding the information on pregnancy and childbirth^34^. A new finding was that the first-time mothers also turned to ‘apps’ for pregnancy and childbirth information—56% rating them as ‘very valuable’^34^ which led to more CS in women. Furthermore, a study on the determinants of CS deliveries of Bangladesh women showed that CSs were more prevalent among women who regularly watched television, where authors postulated that media exposure aware mothers of delivery options^35^.

However, there is also another side of the media influence on childbirth. The media often portrays vaginal delivery as a precarious and painful affair and this perception may have affected women’s opinions in a negative manner, inciting fear and unease^36^. Moreover, because the media is one of the principal sources for women to learn about childbirth^37^, positive information about CSs in the media would encourage women to perceive them as safer and therefore preferable over vaginal deliveries.

A number of factors are hypothesized for the increasing rate of CSs in Bangladesh, including the high rate of adolescent pregnancy (35%), increased rate of late-aged pregnancy (5%), improved household, educational and socio-economic status, and the ongoing dual nutritional burden^38,39^. Another possible explanation for the substantial interactive associations between maternal education and cesarean delivery in medical facilities is that well-educated and affluent women prefer to deliver in comparatively expensive facilities, which may, in turn, be more likely to perform cesarean sections for financial incentives as well as for training purpose of intern doctors^39^.

The discussion above endorses the fact that multiple factors are hypothesized for the gradual increase of CS deliveries worldwide. However, there remains a gap in analyzing elective CS deliveries in developing countries such as Bangladesh. Maternal mortality ratio (MMR) has declined from 434 deaths per 100,000 live births in the year 2000 to 173 per 100,000 live births in 2017 in Bangladesh^40^. To continue this trend, the contribution of elective CS deliveries needs assessment to appropriately determine the necessity of optimal rate of CS in Bangladesh.

Given the differences between emergency and elective CS, the study explored possible reasons for preemptive CSs among women in Bangladesh. Particularity, exposure to information through media, mobile phones and medical expertise before delivery were hypothesized to influence the mode of delivery, apart from household sociodemographic status. Moreover, as the causes of growing CS rates around the globe remain debatable^1^, this study intended to contribute to that literature by identifying the cohort of women in Bangladesh who chose elective CSs over vaginal delivery.

The primary objective of the study was to identify the cohort of women who were most likely to prefer elective CSs in Bangladesh, particularly assessing whether their socioeconomic backgrounds, antenatal care (ANC) along with regular access to media and ownership of mobile devices could impact the choice of elective CS delivery. Geographical analysis was conducted through multiple heat maps of district-wise distribution of service accessibility in the domains of elective CS, media access, mobile-phone ownership, and ANC to explore the spatial characteristics of this practice. Furthermore, the study assessed whether cultural patterns pertaining to interpersonal violence or physical assaults against women were associated with elective CSs in Bangladesh.

## Theoretical framework

In the context of the present study, access to the preferred mode of health services is pivotal to the materialization of elective CS for women in their reproductive years. Levesque et al.^41^ defined the access to healthcare within the domains of seeking, identification, materialization and attainment of resources and healthcare of an individual or a group that is suitable to the demands of their specific care-needs. The current study employed the framework proposed by Penchansky and Thomas^42^ that optimized the notion of access based on the following five dimensions: accessibility, availability, acceptability, affordability, and accommodation. The framework was later amended by Saurman^43^ with the addition of awareness as the sixth dimension that accounts for the knowledge and communication regarding the health-services in question.

**Figure 1:**
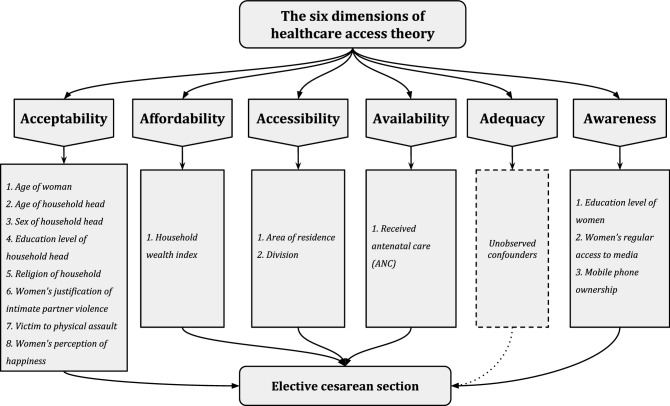
Theory of healthcare access in the context of women’s preference of exercising elective CS during childbirth.

These dimensions are consistent with research that has investigated the utilization of healthcare services as well as the perceptions of the care-seekers regarding these facilities^42^. The selection of applicable variables for this quantitative study hence adhered to the framework of healthcare access theory and the subsequent availability of pertinent variables in the survey data and is illustrated in Fig. 1. However, on account of the unavailability of germane information in the survey data, the dimension of adequacy (accommodation) could not be addressed relating to the practice of elective CSs for women in Bangladesh.

## Results

### Bivariate analyses

Around 20% of women chose to have an elective CS when they delivered their live births during the last 2 years preceding the survey (Table 1). The mean age of women who had an elective CS was 26.2 years. Women with higher levels of education had a greater prevalence of elective CS than women with lower education. Similar statistics were observed for the education levels of household heads. Moreover, the prevalence of elective CS in urban areas (27.8%) was higher than in rural areas (17.8%). For each step increase in the household wealth index quintiles, nearly a 10% increase in elective CS was observed with the richest group (top quintile) having the highest percentage of 38.8%. Khulna and Dhaka divisions had the highest prevalence of elective CSs (30.8% and 27.3%, respectively) compared to other divisions while Chattogram and Sylhet had the lowest two at 12% and 12.8%, respectively. The proportion of elective CSs was higher for women living in a household with a woman house-head than a male house-head.

**Table 1:** The distribution of elective cesarean section (binary) by sociodemographic variables (categorical and continuous) among women who gave birth in the last two years from MICS 2019.

Sociodemographic variables	Group	N (%) of all study participants	N (%) of elective cesarean section	p-value
Education level of woman	Pre-primary/no education	841 (09.1)	42 (5.0)	< 0.001
	Primary	2136 (23.2)	208 (09.7)	
	Secondary	4664 (50.7)	968 (20.8)	
	Higher	1561 (17.0)	594 (38.1)	
Area of residence	Rural	7458 (81.0)	1328 (17.8)	< 0.001
	Urban	1744 (19.0)	484 (27.8)	
Age of woman*		26.0 [5.86]	26.2 [5.36]	0.108**
Household wealth index	Poorest	2212 (24.0)	148 (06.7)	< 0.001
	Poorer	1877 (20.4)	222 (11.8)	
	Middle	1797 (19.5)	354 (19.7)	
	Richer	1761 (19.2)	484 (27.5)	
	Richest	1555 (16.9)	604 (38.8)	
Division	Dhaka	1778 (19.3)	486 (27.3)	< 0.001
	Barishal	821 (08.9)	132 (16.1)	
	Chattogram	1911 (20.8)	229 (12.0)	
	Khulna	1259 (13.7)	388 (30.8)	
	Mymensingh	552 (06.0)	76 (13.8)	
	Rajshahi	950 (10.3)	209 (22.0)	
	Rangpur	1129 (12.3)	189 (16.7)	
	Sylhet	802 (08.7)	103 (12.8)	
Age of household head*		42.6 [14.65]	44.1 [14.91]	< 0.001**
Sex of household head	Male	8573 (93.2)	1669 (19.5)	0.047
	Female	629 (06.8)	143 (22.7)	
Education level of household head	Pre-primary/no education	2792 (30.3)	321 (11.5)	< 0.001
	Primary	2776 (30.2)	429 (15.5)	
	Secondary	2586 (28.1)	649 (25.1)	
	Higher	1048 (11.4)	413 (39.4)	
Religion of household head	Muslim	8311 (90.3)	1628 (19.6)	0.449
	Others	891 (9.7)	184 (20.7)	
Women’s regular access to media	No access	3777 (41.0)	393 (10.4)	< 0.001
	Has access	5425 (59.0)	1419 (26.2)	
Mobile ownership	Does not own	2242 (24.4)	250 (11.2)	< 0.001
	Owns	6960 (75.6)	1562 (22.4)	
Women's justification of intimate partner violence (IPV)	Not justified	6846 (74.4)	1479 (21.6)	< 0.001
	Justified	2356 (25.6)	333 (14.1)	
Victim to physical assault	Not victimized	8783 (95.4)	1749 (19.9)	0.014
	Victimized	419 (04.6)	63 (15.0)	
Women's perception of happiness	Not happy	1118 (12.1)	113 (10.1)	< 0.001
	Happy	8084 (87.9)	1699 (21.0)	
Received antenatal care (ANC)	Did not receive care	1662 (18.1)	63 (03.8)	< 0.001
	Received care	7540 (81.9)	1749 (23.2)	
Total sample size		9202	1812 (19.7)	

*Mean [standard deviation].

**p-value from t-test.

Women who had regular access to the media (newspaper, television, radio, and internet) showed an increased likelihood of opting for an elective CS (26.2%) than women who had no such access (10.4%). Similarly, mobile ownership also raised the prevalence by nearly 11%. Women justifying IPV perpetrated by their husbands reported a lower percentage of elective CS and the same applied to women who were physically assaulted. Unhappier women showed a decreased tendency of opting for an elective CS (10.1%) compared to the overall prevalence. Lastly, only 3.8% of women who did not receive antenatal care (ANC) during pregnancy opted for an elective CS compared to women who received ANC (23.2%).

### Generalized linear model

Age of women was positively associated with elective CS with the adjusted odds ratio (AOR) at 1.02 (p-value < 0.001, Table 2). In comparison to women with no education or only pre-primary education, secondary or higher educated women were respectively 2 times and 3 times more likely to choose an elective CS (AOR 2.15 and 3.17 with p-value < 0.001). Similarly, women living in households with secondary or higher educated house-heads had respectively 43% and 63% more chances of an elective CS (p-value < 0.001) compared to those with no or pre-primary educated house-heads. Women living in wealthier households (middle, richer, and richest) had a substantially increased likelihood of elective CS compared to women living in underprivileged households.

**Table 2:** Generalized Linear Model (GLM) fitted to binary outcome variable ‘elective cesarean section’ (binary) with sociodemographic variables adjusting for cluster and strata-wise variations and survey weights.

Sociodemographic variables	Elective cesarean-section AOR (95% CI)	p-value
Age of woman	1.02 (1.01, 1.04)	< 0.001
**Education level of woman (ref: pre-primary/no education)**		
Primary	1.43 (0.95, 2.14)	0.084
Secondary	2.15 (1.46, 3.17)	< 0.001
Higher	3.17 (2.10, 4.79)	< 0.001
**Household wealth index (ref: poorest)**		
Poorer	1.25 (0.96, 1.62)	0.103
Middle	1.75 (1.35, 2.27)	< 0.001
Richer	2.12 (1.62, 2.79)	< 0.001
Richest	3.04 (2.24, 4.12)	< 0.001
**Area of residence (ref: rural)**		
Urban	0.92 (0.77, 1.09)	0.321
**Division (ref: Dhaka)**		
Barishal	0.68 (0.52, 0.89)	0.005
Chattogram	0.44 (0.36, 0.55)	< 0.001
Khulna	1.21 (0.99, 1.47)	0.064
Mymensingh	0.83 (0.62, 1.12)	0.220
Rajshahi	1.01 (0.81, 1.25)	0.950
Rangpur	0.81 (0.63, 1.03)	0.086
Sylhet	0.59 (0.43, 0.80)	0.001
**Sex of household head (ref: male)**		
Female	1.07 (0.83, 1.37)	0.618
Age of household head	1.01 (1.00, 1.01)	0.021
**Education level of household head (ref: pre-primary/no education)**		
Primary	1.13 (0.93, 1.38)	0.212
Secondary	1.43 (1.18, 1.74)	< 0.001
Higher	1.63 (1.27, 2.09)	< 0.001
**Religion of household head (ref: others)**		
Muslim	0.80 (0.64, 1.00)	0.051
**Women’s regular access to media (ref: no access)**		
Has access	1.31 (1.10, 1.56)	0.003
**Mobile ownership (ref: does not own)**		
Owns	1.25 (1.04, 1.50)	0.020
**Women's justification of intimate partner violence (IPV) (ref: not justified)**		
Justified	0.89 (0.76, 1.04)	0.147
**Victim to physical assault (ref: not victimized)**		
Victimized	0.99 (0.73, 1.34)	0.943
**Women's perception of happiness (ref: not happy)**		
Happy	1.28 (1.00, 1.63)	0.051
**Received antenatal care (ANC) (ref: did not receive care)**		
Received care	4.12 (3.06, 5.54)	< 0.001

*AOR* adjusted odds ratio, *CI* confidence interval.

In comparison to the Dhaka division, women living in Sylhet and Chittagong showed a lower likelihood of opting for elective CS (AOR 0.44 and 0.59, respectively). Older house-heads seemed to increase the likelihood of choosing elective CSs with a 1% higher odds for each year increase in age. Muslim women were 20% less likely to have an elective CS compared to women belonging to other religions (p-value = 0.051). Women’s regular access to media was significantly associated with opting for an elective CS (AOR 1.31 with p-value 0.003). Similarly, ownership of a mobile phone (AOR 1.25 with p-value 0.020) was also positively associated with an elective CS. Happier women seemed to prefer an elective CS (AOR 1.28 with p-value = 0.051). Lastly, women who received antenatal care (ANC) were more than four times as likely to choose an elective CS (AOR 4.12 with p-value < 0.001) compared to women who did not receive ANC during pregnancy.

The Generalized Variance Inflation Factor (GVIF) scores were assessed to identify any presence of multicollinearity in the model where the threshold value for the squared adjusted GVIFs for categorical variables are equivalent to the conventional VIF threshold for continuous variables^44^. As all squared adjusted GVIFs were less than 5, thus we could conclude that there was no multi-collinearity in the model. These are detailed in the table within the supplementary file (Supplementary Table 1).

### Spatial mapping

**Figure 2:**
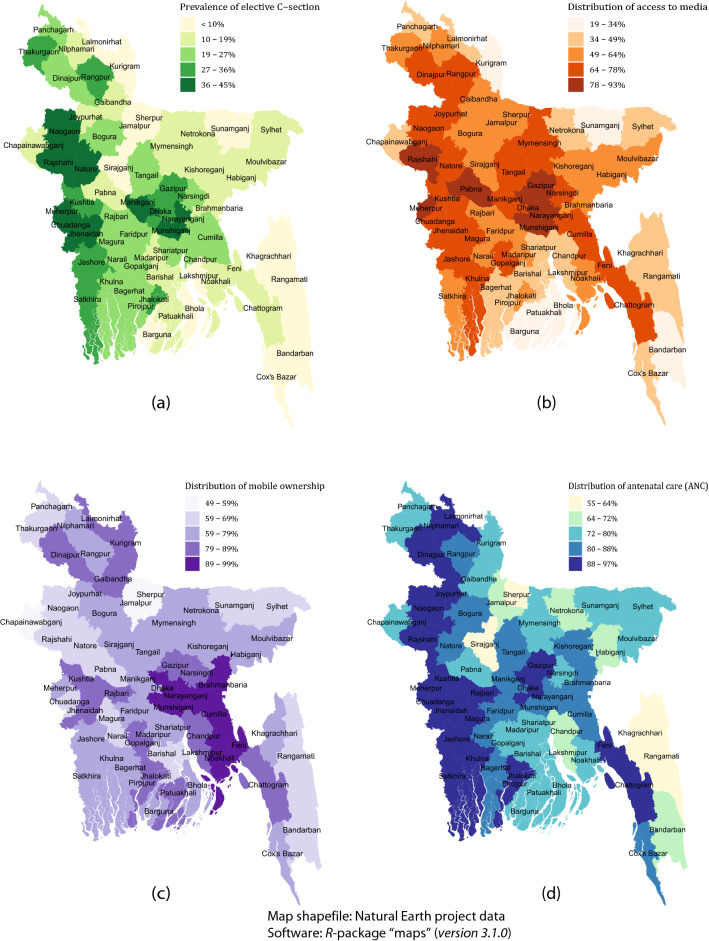
District-wise spatial mapping on the distribution of (**a**) elective CS, (**b**) access to media, (**c**) mobile ownership, and (**d**) antenatal care coverage.

The district-wise spatial distribution of elective CSs among women in Bangladesh, depicted in Fig. 2a, was evaluated in order to obtain further insights regarding this practice. It was observed that the western part of the country, primarily Rangpur, Rajshahi, and Khulna as well Dhaka divisions, had the highest proportions of opting for elective CSs, corroborating the previous findings. The highest prevalence of elective CSs was found in the Meherpur district at 45.3% of all live births (53 out of 117 live births) 2 years prior to the survey while Bandarban had the lowest prevalence at 0.7% (1 out of 134 live births).

Fewer than 50% of women in the border areas of the northern and southern parts of the country had regular access to any form of media (Fig. 2b). For media access, the highest percentage of opting elective CS was reported in Narayanganj at 93.1% and the lowest was reported in the island-district of Bhola at 19.4%. Nearly 50% or more women around the country reported that they owned a mobile phone (Fig. 2c) with Narayanganj reporting the highest percentage (98.5%) and Sherpur with the lowest (48.6%).

The spatial distribution of ANC coverage in Fig. 2d is similar to elective CS prevalence shown in Fig. 2a. The highest coverage of ANC was found in the district of Kushtia (96.6%) and the lowest was in Sirajganj (55.4%). Elective CS was higher in the western part of the country which includes Rangpur, Rajshahi and Khulna divisions along with Dhaka where ANC coverage was also high.

### Classification tree

**Figure 3:**
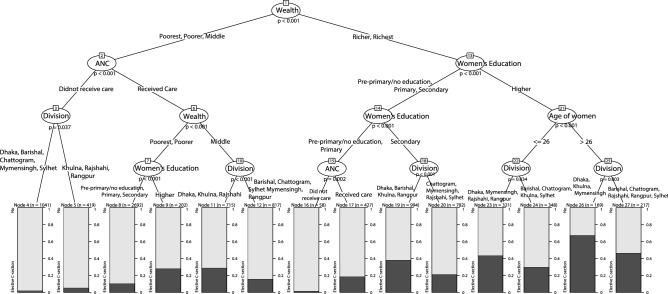
Classification tree diagram displaying the important predictors of elective CS for women in Bangladesh.

The classification tree showed that household wealth, corresponding to the dimension of affordability of the theory of healthcare access^42^, was the most important predictor of elective CSs (Fig. 3). On the left-hand branch from the top node, that is, among the lower three quintiles of wealth index, the prevalence of opting for elective CSs were low across the two nodes (node 4 and 5) where the women did not receive ANC. Node-9 shows that even if women are from the poorer or poorest households, if they received ANC and had a higher level of education, the chance of opting for an elective CS during childbirth was 28.2%, whereas, for the same group with an education level of secondary or lower (node-8) this proportion was less than 10%. Furthermore, in node-11, women living in the middle-category household wealth who received ANC, and living in Dhaka, Rajshahi and Khulna divisions were more likely to prefer elective CSs than the women living in other divisions of the same group.

On the right-hand branch of the tree, node 26 shows the highest prevalence of elective CS at 67.5%. That is, higher educated women, aged 26 or higher, living in richer or richest households and residing in Dhaka, Khulna and Mymensingh divisions hold the highest percentage of opting for elective CSs, while the prevalence for women in other divisions with the same profile is 46.5%, which was still considerably high.

## Discussion

The findings suggest that educated women living in affluent households with educated house heads, who accessed ANC prior to delivery with regular access to media, including mobile phones complemented by geographical area of residence tend to prefer elective CS deliveries in Bangladesh. Overall, health access and health literacy seem to play key roles in choosing the mode of delivery. While the debate on excessive use of CS, will continue for the reasons stated above, the findings from this study suggest that health awareness, health education and better health communication via mass media would allow mothers to access appropriate healthcare advice on options for a safe birth and related issues. The overall CS delivery scenario includes medically-induced CS deliveries that are typically suggested by the medical professionals at the time of delivery and sometimes medical facilities tend to impose unnecessary CS delivery on the patients justifying medical reasons^45^. Since this study limited itself to addressing only the elective CS deliveries opted by the mothers and not compared against other forms of delivery, this overall debate regarding excessive CS prevalence thus falls out of the scope. Policymakers should consider the major contributing factors, such as media exposure and pre-delivery care, as interventions for future regulation regarding maternal reproductive health care.

Bangladesh has achieved an unprecedented level of success in public health over the past two decades^46^. Starting from vaccination coverage to foreign direct investment in local non-governmental institutions, these efforts have allowed access to multifaceted maternal healthcare in peripheral areas of Bangladesh^47,48^. However, informed healthcare decision-making requires the care recipients to be able to weigh their options and make the safest choice, which demands enhanced health communication strategies and greater reach to women from all communities^49,50^. It has been found that the media could be a cost-effective process of disseminating information on CSs. Such promotions have previously been found to be effective awareness tools for health education in regions with low literacy rates, such as Bangladesh, as these would encourage mothers to consult and take appropriate advice from medical professionals^51^.

Similar to media exposure, ownership of mobile phones creates an avenue for health promotion with further increases exposure to information media. The Government in Bangladesh regularly sends text messages on various issues, and women could be direct recipients of these if they own a personal mobile phone^51–53^. Direct access to health information has multi-dimensional impacts. First, it allows the health providers to be in contact with the mothers and cater to their urgent needs, especially in remote areas of Bangladesh where access to healthcare facilities is inadequate^54,55^. Second, given that the duration of pregnancy is nine months, access to a medium of communication allows mothers to regularly discuss their delivery options with both family and health experts^56,57^. Third, awareness through media or mobile phones could be an effective mechanism in a patriarchal society such as Bangladesh, where knowledge about delivery options would empower women encouraging them to seek professional advice regarding delivery options^58,59^.

A key element to successful delivery during childbirth is antenatal care. In Bangladesh, women who received antenatal care were shown to have greater access to skilled delivery care, which suggested that antenatal care encouraged women and their families to access modern services in health facilities^60^. Similar reasoning and outcomes are expected in the case of delivery options, as skilled community health workers would provide the necessary delivery options, including CSs, to the prospective mothers to lessen the chance of complications^61,62^. Thus, access to such care would allow health professionals to give the best delivery care for mothers, instead of families or mothers making a decision based on traditions or unregulated private sector advertisements. Accessing maternal healthcare will reduce the unnecessary CS deliveries as well as render this delivery option to those who need it.

The spatial analyses observed a higher density of CS deliveries in the central and western parts of Bangladesh, primarily in Dhaka, Rajshahi, Khulna and the adjacent districts. Similar patterns were found for media exposure and ANC. This analysis supported the initial hypothesis that distribution of information and access to healthcare led to higher numbers of CS deliveries. The policy focus here would be to extend ANC care in peripheral districts such as Sirajganj, Sherpur, and the hill tracts of Chittagong. Both media and mobile phone access need to be increased countrywide to improve the health literacy of women in remote areas so that mothers can access appropriate maternal healthcare and delivery advice.

Higher sociodemographic conditions were found to be associated with elective CSs. These conditions corroborated the existing literature, as cesarean sections require institutional facilities that demand financial solvency^39,63^. Profit-driven private facilities have increased the proportion of CSs as they provide better care, and are more likely to be frequented by affluent educated classes living in metropolitan areas^64,65^. Similarly, educated women are more likely to consider the ‘safer’ CS option in comparison to the normal vaginal delivery^30,66^. While these demographic factors were hypothesized to be associated with elective CSs, contrary to the literature the outcomes of IPV were not found to be associated with elective CSs^67^.

There were a small number of limitations to the study. First, the respondents were subjected to recall bias; for example, for a pregnancy dating back 2 years, any information on IPV during pregnancy could be not verified. Second, cross-sectional data were used for the analysis, which limited the conclusion from making any causal inference. Third, data on delivery locations, especially a categorization of private and public health facilities, would have provided a greater depth to the current findings, which could not be done due to the secondary nature of the data. Future data collection efforts could consider including these details. Fourth, detailed information on the facilities and motivations behind elective CSs would have allowed a greater interpretation of the findings. However, this study provided a platform for future longitudinal studies that can fill the gaps identified.

The first target of Sustainable Development Goal 3.1 is to reduce the maternal mortality ratio to less than 70 per 100 000 live births by 2030^68^. For Bangladesh to achieve this target, it must ensure the availability of safe delivery options to mothers. Some issues are to disseminate maternal information to mothers, increase the countrywide health literacy rate, and enlighten women of reproductive ages regarding their choices of delivery methods such as vaginal birth or CS delivery. The study identified that women with access to media and mobile phones seemed to have a greater scope at choosing elective CS delivery, suggesting that these could be an avenue for the promotion of reproductive health. In addition, antenatal care or pre-delivery care also allowed mothers to opt for CSs, demonstrating the impact of skilled community healthcare.

While there could be a debate on the necessity of nearly 20% elective CS deliveries in Bangladesh, there is also a need to discuss the choices of delivery methods a mother is provided with before childbirth. This study found that deliveries in remote districts, particularly those that were far from divisional cities, were less likely to have elective CS deliveries. The overconcentration of elective CSs in the metropolitan cities suggested that more health promotion and facility accessibility in peripheral areas were required as well as an investigation in areas with an excessive density of CS deliveries. A focused analysis of these issues could reveal further insights regarding maternal healthcare and women’s access to delivery options. Policymakers in Bangladesh should direct their focus on multiple fronts if Bangladesh is to reach the SDG goal 3.1 by 2030.

## Methods and materials

### Data overview

The study analyzed the sixth round of the nationally representative Bangladesh Multiple Indicator Cluster Survey (MICS) 2019 data. The survey employed a two-stage stratified cluster sampling method where each of the 64 districts was considered as the sampling strata^69^. The primary sampling units were the enumeration areas (EAs) based on the 2011 Bangladesh population and the secondary sampling units were housing census and households. In the first stage of sampling, a total of 3220 EAs were selected using the probability proportional to size (PPS) method from all 64 strata. A random systematic selection was used in the second stage to select a sample of 20 households from each sampled EA yielding a final sample of 64,400 households^25^.

The present analyses considered women in their reproductive years with a live birth in the past two years prior to the survey. The working dataset was constructed by combining the individual datasets on women aged 15–49 years and their respective household characteristics. After cleaning the data of missing values, the final sample size for the study was 9202.

### Ethical clearance

This study was based on analysis of a secondary survey data from UNICEF, where all the personally identifiable information of participants had been removed. Informed consent was taken from participants before participating in the survey by the national statistical office, Bangladesh Bureau of Statistics and UNICEF. The data are available online: http://mics.unicef.org/surveys.

### Independent variables

The sociodemographic characteristics of the women along with their household information were taken into account based on the theoretical framework, where the variables corresponded to five dimensions (accessibility, availability, acceptability, affordability, and awareness) out of the six dimensions of healthcare access theory^43^. Age of women (respondent), as well as age, sex, religion, and education level of household heads belonged to the dimension of acceptability of access which corresponded to the social and cultural aspects of the individuals. Furthermore, the binary variables ‘women's justification of intimate partner violence (IPV)’, ‘victimization to physical assault’, and ‘women's perception of happiness’ belonged to the dimension of acceptability.

The binary variable women's justification of IPV (justified, not justified) was simulated based on the affirmative answer to any one of the questions that asked whether the respondent considered IPV justified if her husband beat her for going out without seeking permission from her husband, neglected the children, argued with her husband, burnt food, or refused to have sex with husband. Victimization in relation to physical assault (victimized, not victimized) was coded based on whether the woman was physically abused at home or outside and was extracted directly from the survey data. Women's perception of happiness (happy, not happy) was based on the answers to the question that assessed how happy the woman was with her life. If she responded very happy or somewhat happy, it was coded as ‘happy’ and otherwise ‘not happy’.

Affordability, reflecting the financial capability of women to access the desired healthcare and services, was addressed by the household wealth index quintile, which is a predefined variable in the survey based on household assets. Area of residence and administrative division corresponded to the dimension of accessibility that embodied the spatial aspects of access. Lastly, the education level of women, their regular access to media and ownership of mobile phones addressed the final dimension of awareness that involves the knowledge and communication aspects of the healthcare services^42,43^. The four variables that corresponded to using newspaper, radio, television, and the internet were recoded into the binary variable ‘women’s regular access to media’ (has access, no access), where women who responded affirmatively to using at least one of these forms of media at least once a week or every day were considered in the ‘has access’ category and the rest belonged to the ‘no access’ category.

### Outcome variables

The binary outcome variable was whether the respondent had an elective CS within the last 2 years of the survey. If the decision for having a CS was taken before the labor began, the childbirth was considered a birth by elective CS and was coded ‘yes’ and otherwise ‘no’^25^.

### Statistical analyses

The statistical analyses were conducted in four stages. First, bivariate analyses including the chi-square tests of independence and t-tests (where appropriate) were carried out to evaluate the primary associations between the sociodemographic variables and the outcome variable^70^. Second, a generalized linear model (logistic regression model) for the binary outcome was fitted to analyze the multivariable associations between the study variables and the outcome after adjusting for the cluster and strata-wise variations and survey weights to generalize the findings^71^.

Third, the spatial distributions of the variables of importance were mapped to investigate their distributions across 64 districts of Bangladesh. The spatial distribution of the CS deliveries corresponds to the dimension of accessibility and is integral to the understanding of the inequality in delivery options for women around the country. Finally, the interpretable machine learning method of building classification trees^72^ was used. For model sensitivity, the squared adjusted generalized variance inflation factor (GVIF) scores were quantified to assess multicollinearity in the model^44^.

Classification trees are part of machine learning techniques where the sample data are divided into multiple subgroups, called nodes, based on simple rules involving the predictors using binary recursive partitioning. It is non-parametric in nature and offers easier interpretations of the complex non-linear interactions of variables in high-dimensional settings^72^. The present analyses utilized the classification tree to identify the multifactor interactions of significance between the sociodemographic predictors of elective CS in Bangladesh. Here, the conditional inference framework proposed by Hothorn et al.^73^ was applied, thus avoiding much of the criticism typically received by standard classification tree techniques. While the trees offer flexible approaches to identify interactions among explanatory variables from the sample, they do not provide measurable sizes of effects of these interactions on the response. Therefore, the combined results of classification tree and the logistic regression model were interpreted and elaborated in the discussion section.

All analyses were conducted in R (version 3.6.0). The binary logistic regression model (GLM) was fitted using the “survey” package (version 3.37). Natural Earth project data^74^ was used through the R-package “maps” (version 3.1.0) to construct the district-wise heat maps^75^. Finally, the package “partykit” (version 1.2-7) was utilized to obtain the classification tree based on the conditional framework approach^76^.

## Supplementary Information

Supplementary Information.
