# Supplementary material for: A national assessment of elective cesarean sections in Bangladesh and the need for health literacy and accessibility
Source: Sci Rep. 2021 Aug 19;11:16854. doi: 10.1038/s41598-021-96337-0 (PMC8376956; doi:10.1038/s41598-021-96337-0)
Supplement: Supplementary file 1 — Supplementary Information. [file 41598_2021_96337_MOESM1_ESM.docx]

**Elective cesarean sections in Bangladesh: A need for health promotion and health literacy among women**

**Awan Afiaz**

Institute of Statistical Research and Training, University of Dhaka, Dhaka, Bangladesh

[aafiaz@isrt.ac.bd](mailto:aafiaz@isrt.ac.bd)

**Anowara Rayhan Arusha**

Applied Linguistics and English Language Teaching (ELT), University of Dhaka, Bangladesh

[a.rayhan.arusha@gmail.com](mailto:a.rayhan.arusha@gmail.com)

**Nurjahan Ananna**

Ibrahim Medical College, Dhaka, Bangladesh

[nurjahansayed.ananna@gmail.com](mailto:nurjahansayed.ananna@gmail.com)

**Enamul Kabir**

School of Sciences, University of Southern Queensland, Toowoomba, Queensland, Australia

Enamul.Kabir@usq.edu.au

**Raaj Kishore Biswas**

Transport and Road Safety (TARS) Research Centre, School of Aviation, University of New South Wales, Australia

[RaajKishore.Biswas@student.unsw.edu.au](mailto:RaajKishore.Biswas@student.unsw.edu.au)

Supplementary Table 1: The generalized variance inflation factors associated with the regressors in the generalized linear regression model (GLM).

| Variables in the model | GVIF | Degrees of freedom | Adjusted GVIF = GVIFˆ(1/(2*DF)) | **Squared Adjusted GVIF** |
| --- | --- | --- | --- | --- |
| Age of woman | 1.16 | 1 | 1.08 | 1.16 |
| Education level of woman | 1.91 | 3 | 1.11 | 1.24 |
| Wealth index | 2.65 | 4 | 1.13 | 1.28 |
| Area of residence | 1.45 | 1 | 1.21 | 1.45 |
| Division | 1.68 | 7 | 1.04 | 1.08 |
| Sex of household head | 1.12 | 1 | 1.06 | 1.12 |
| Age of household head | 1.21 | 1 | 1.1 | 1.21 |
| Education level of household head | 2.02 | 3 | 1.12 | 1.26 |
| Religion of household head | 1.07 | 1 | 1.03 | 1.07 |
| Women's regular access to media | 1.44 | 1 | 1.2 | 1.44 |
| Mobile Ownership | 1.15 | 1 | 1.07 | 1.15 |
| Women's justification of intimate partner violence (IPV) | 1.08 | 1 | 1.04 | 1.08 |
| Victim to physical assault | 1.12 | 1 | 1.06 | 1.12 |
| Women's perception of happiness | 1.12 | 1 | 1.06 | 1.12 |
| Received antenatal care (ANC) | 1.10 | 1 | 1.05 | 1.10 |
